# Characterization of LE3 and LE4, the only lytic phages known to infect the spirochete *Leptospira*

**DOI:** 10.1038/s41598-018-29983-6

**Published:** 2018-08-06

**Authors:** Olivier Schiettekatte, Antony T. Vincent, Christian Malosse, Pierre Lechat, Julia Chamot-Rooke, Frédéric J. Veyrier, Mathieu Picardeau, Pascale Bourhy

**Affiliations:** 1 Institut Pasteur, Unité Biologie des Spirochètes, Paris, France; 2 INRS-Institut Armand-Frappier, Bacterial Symbionts Evolution, Laval, Quebec, Canada; 3 Institut Pasteur, Citech, Mass Spectrometry for Biology Utechs, USR, 2000 IP CNRS Paris, France; 4 Institut Pasteur, Bioinformatics and Biostatistics Hub, C3BI, USR, 3756 IP CNRS Paris, France

## Abstract

*Leptospira* is a phylogenetically unique group of bacteria, and includes the causative agents of leptospirosis, the most globally prevalent zoonosis. Bacteriophages in *Leptospira* are largely unexplored. To date, a genomic sequence is available for only one temperate leptophage called LE1. Here, we sequenced and analysed the first genomes of the lytic phages LE3 and LE4 that can infect the saprophyte *Leptospira biflexa* using the lipopolysaccharide O-antigen as receptor. Bioinformatics analysis showed that the 48-kb LE3 and LE4 genomes are similar and contain 62% genes whose function cannot be predicted. Mass spectrometry led to the identification of 21 and 23 phage proteins in LE3 and LE4, respectively. However we did not identify significant similarities with other phage genomes. A search for prophages close to LE4 in the *Leptospira* genomes allowed for the identification of a related plasmid in *L. interrogans* and a prophage-like region in the draft genome of a clinical isolate of *L. mayottensis*. Long-read whole genome sequencing of the *L. mayottensis* revealed that the genome contained a LE4 phage-like circular plasmid. Further isolation and genomic comparison of leptophages should reveal their role in the genetic evolution of *Leptospira*.

## Introduction

Recently, there has been a renewed interest in bacteriophages for their potential use as alternatives to conventional antibiotics^1^, and also in understanding their contribution in evolution of bacteria^2,3^. In addition, phages could be used to develop new genetic tools such as replicative vectors from different compatibility groups and phage-delivery systems. Unfortunately, little is known about the diversity of phages among the genus *Leptospira*. *Leptospira* are ubiquitous organisms that are found as free-living saprophytes in environmental water and soil, or as pathogens that can cause acute or chronic infections in animals. A third group that is composed of intermediate species (in regards to their pathogenesis) of *Leptospira*, is phylogenetically closely related to the pathogenic species and can cause mild infections^4^. Leptospirosis is an emerging waterborne zoonosis which results in more than one million human cases a year with a fatality rate frequently exceeding 10%^5^.

To the best of our knowledge, the only phages that have been isolated, purified, and phenotypically characterized in the genus *Leptospira* are: vB_LbiM_LE1 (renamed^6^, and abbreviated LE1), vB_LbiM_LE3 (LE3), and vB_LbiM_LE4 (LE4)^7^. These tailed phages have been isolated from urban sewage and infect the saprophyte *L. biflexa*. The LE1 temperate phage genome was previously sequenced^8^ and an *Escherichia coli-L. biflexa* shuttle vector was generated by cloning the replication origin of LE1^9^. Until now, the virulent phages LE3 and LE4 have not been further characterized at the genomic and proteomic levels. In addition to these three phages, phage-like particles were also observed following mitomycin C induction of a pathogenic strain carrying a circular plasmid with phage-related genes, but these phage-like particles were not purified^10^.

Recently, comparative analyses of genome sequences have suggested the existence of prophages and genomic islands within the genus *Leptospira*^11,12^. Putative prophages are found in infectious *Leptospira* species, including pathogenic and intermediate species, and absent in saprophytic *Leptospira* species, suggesting that phages may have had a major role in the emergence of the pathogens and/or in the acquisition of virulence factors. Analysis of the complete genomes of 20 *Leptospira* species led to the description of several predicted prophage regions^11^, including LE1-like and Mu-like prophages. In addition, the existence of two other groups of prophages have been proposed: a 22-kb region which was initially described in *L. interrogans* serovar Lai but that is present in most pathogenic species and the intermediate species *L. licerasiae*^11–13^ and a 65-kb region, observed in *L. interrogans* serovar Manilae, which can excise from the chromosome and form a circular replicon^14,15^ that is closely related to a 70-kb circular plasmid, pLIMLP1^16^.

Characterizing leptophages—phages that infect *Leptospira*—sequencing their genomes, identifying major proteins, understanding their genetic relationships, and studying their interactions with various host strains are all essential steps in the understanding of *Leptospira* evolution. In addition, most replicative vectors are based on the replication systems derived from phage or prophage-like elements^17^. These vectors are used for genetic complementation, heterologous expression and controlled gene expression. However, pathogenesis research is slowed down by the lack of practical tools for routine genetic manipulation of pathogenic *Leptospira* strains. For all these reasons, we report herein the characterization of the first virulent leptophages at the phenotypic, genomic, and proteomic levels. We also demonstrate that these leptophages use lipopolysaccharides (LPS) as a receptor on bacterial cells. A general analysis of LE3 and LE4 genomes did not reveal significant similarities with other viral genomes, suggesting they may constitute a new group of phages. We also searched for related prophages in *Leptospira* genomes available in public databases, leading to the identification of novel circular plasmids in a clinical isolate.

## Results and Discussion

### Morphology

**Figure 1:**
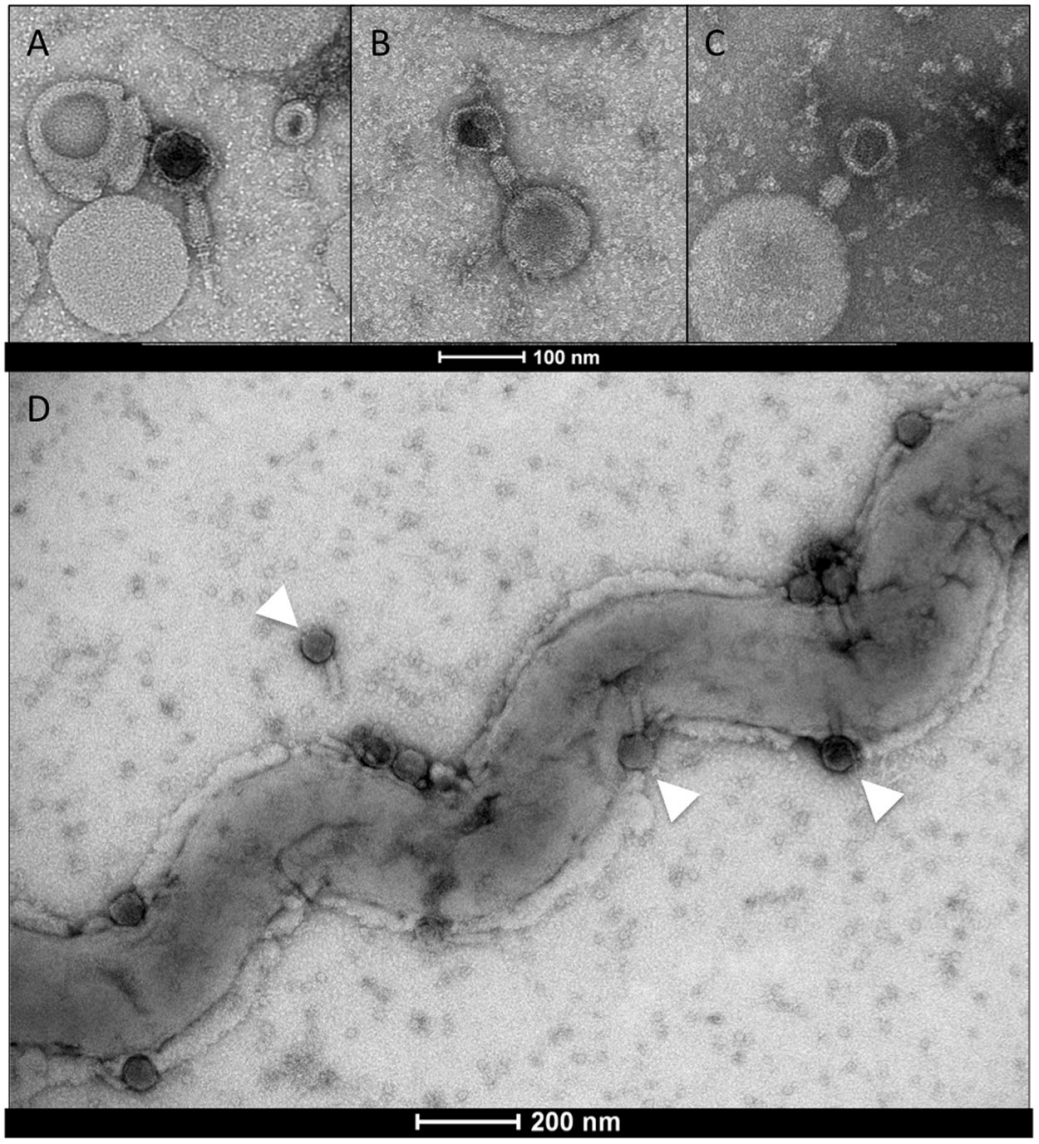
Transmission electron micrographs of leptophages. Morphology of leptophages (**A**) LE1, (**B**) LE3, and (**C**) LE4. Scale bar represents 100 nm. (**D**) Magnification of *L. biflexa* strain Patoc 1 (cell length of 10 µm) six hours after infection with phage LE4 (MOI=10). Arrowheads indicate representative phages. Scale bar represents 200 nm.

Three tailed phages, designated LE1, LE3, and LE4, were previously isolated from urban sewage by direct plating of water samples using *L. biflexa* as a host^7^. Cultures of *L. biflexa* were inoculated with LE3 and LE4 at a multiplicity of infection (MOI) of 0.001, showing a moderate lysis—up to 50%—after one month. Purified viruses observed by electron microscopy share the same common tailed-phage morphology consistent with their affiliation with the *Myoviridae* family (from the order *Caudovirales*) which is one of the most represented and characterized in bacteriophages^18^. *Myoviridae* cannonically possess tails that mechanically contract during infection. LE1 has a hexahedral capsid of 80 nm in diameter, and a contractile tail of 75 to 115 nm in length (Fig. 1A, n = 15 phages measured). LE3 and LE4 have a hexahedral capsid of 65 nm in diameter, and a contractile tail of 65 to 80 nm in length (Fig. 1B–D n = 12 and n = 20 phages measured). Most of the LE3 and LE4 particles were found to be associated with membrane-derived vesicles. It is actually unclear if these vesicles are artifacts due to the lysis or if they are produced by the bacterial cells to serve as decoys, as previously reported for *E. coli*^19^.

To better characterize the lytic phage infectious cycle, *L. biflexa* was challenged with LE4 at an MOI of 10 and the cells were observed by electron microscopy at different times (Supplementary Fig. S1). At two hours post-infection, approximately two phages are attached per cell. This small number of cell-associated phage particles may be due to poor adsorption of phages, vesicles and remnants of cells adsorbing phages, or loss of phages during the grid preparation. Six hours post-infection, cells start to release LE4 virions. An electron micrograph shows the release of at least 25 phage particles along the length (10 µm) of the infected cell (Fig. 1D). This number is most likely underestimated, as additional particles are probably being assembled and matured within the host cell or not visible in the two-dimension micrographs. The phage particles seem to exit through the cell envelop without lysing the whole infected cell, at least during the early stage of the burst. At 24 hours, infected cultures present phage particles, cell remnants and rare intact cells (Supplementary Fig. S1).

### Phage absorption and one-step growth kinetics

The adsorption behaviour of LE4 to *L. biflexa* strain Patoc 1 was studied at a MOI of 0.0001. Approximately 20% of the phages were adsorbed in 30 min and the adsorbed phages reached a maximum of 70% after 3 h of incubation (Supplementary Fig. S2).

**Figure 2:**
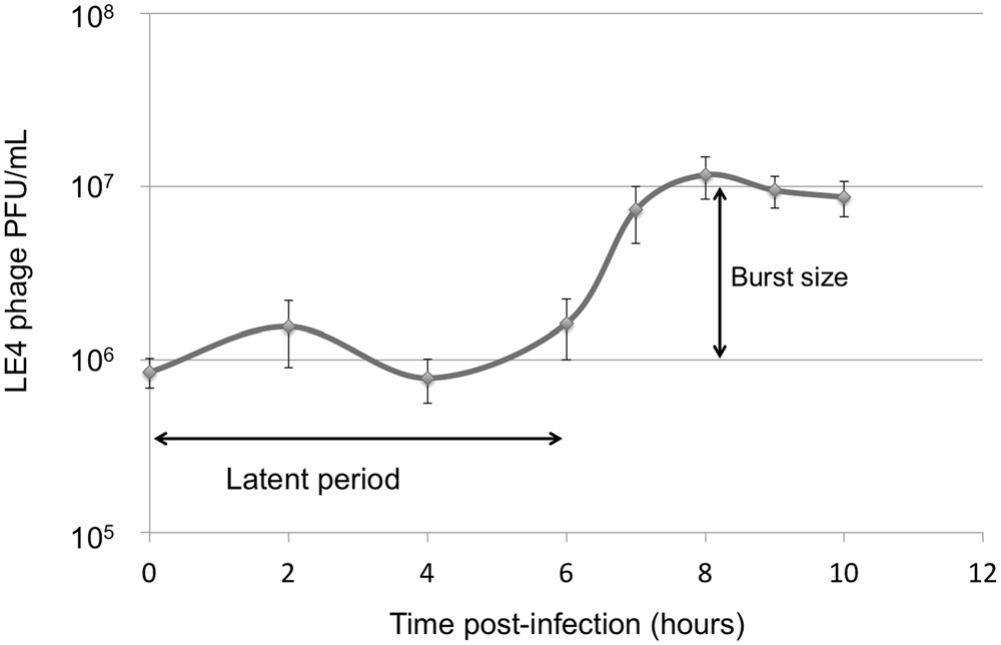
One-step growth curve of LE4 infecting *L. biflexa*. Data from six experiments; error bars indicate standard deviation.

One-step growth studies were also conducted to investigate the different phases of the phage infection. We then determined the latent period and burst size of LE4 on *L. biflexa*, which are relatively slow growing bacteria, with a doubling time of about 24 hours. According to the one-step growth experiments, the latent period of LE4 on *L. biflexa* was approximately 6 hours and the rise period was 2 hours. The burst size corresponds to ten phages per infected cell (Fig. 2).

### Host range of lytic phages LE3 and LE4

For the determination of the LE3/LE4 host range, 25 strains belonging to 10 *Leptospira* species (*L. biflexa*, *L. interrogans*, *L. borgpetersenii*, *L. kirschneri*, *L. weilii*, *L. noguchii*, *L. meyeri*, *L. yanagawae*, *L. fainei*, and *L. licerasiae*) were tested by spot assays. Lysis activity was limited to the *L. biflexa* strain Patoc 1, and the *L. meyeri* strain 201601301, supporting the hypothesis that these phages have a narrow host range^7^, even though the host strains do not belong to the same species and have been isolated in different geographical regions (Europe and Indian Ocean).

As expected for lytic (virulent) phages, plaques of LE3 and LE4 on lawns of *L. biflexa* formed clear and non-turbid zones of heterogenous sizes ranging from 0.3 mm to 3 mm, with no colonies growing inside the zone of clearance even after prolonged incubation (Supplementary Fig. S3). Moreover, isolated bacterial clone resistant to LE4 were further tested for the presence of LE4 by PCR (see below). We were unable to detect LE4 DNA via colony PCR, further suggesting that the phages LE3 and LE4 are lytic phages and not lysogenic.

### Selection, identification, and characterization of phage resistant mutants

**Figure 3:**
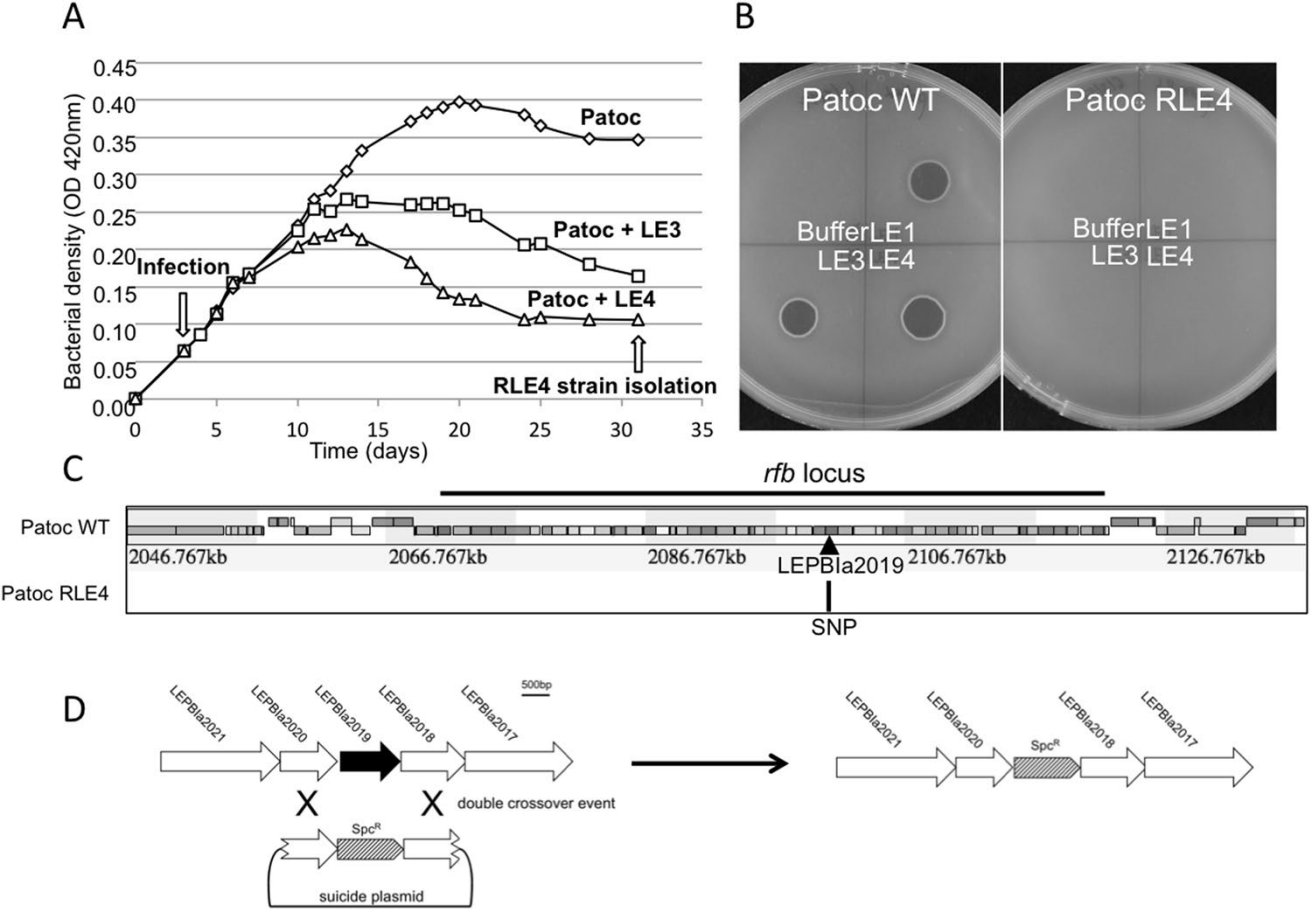
LPS as a receptor of the leptophages LE1, LE3, and LE4. (**A**) Growth curves of *L. biflexa* infected by LE3 and LE4. (**B**) Plaque assay of LE1 LE3 and LE4 (10^3^ PFU) on lawn of *L. biflexa* strain Patoc 1 and *L. biflexa* strain RLE4. (**C**) Genetic map of the LPS locus. The location of SNP is noted on the RLE4 map as an arrow. (**D**) Schematic representation of targeted mutagenesis of LEPBIa2019 in WT *L. biflexa*.

Phages infect bacteria by attaching to surface-exposed receptors and injecting their DNA into the cells. In Gram-negative bacteria, outer membrane proteins, pili, flagella, oligosaccharides, and LPS have all been characterized as phage receptors^20,21^. To better understand the genetic factors influencing bacterial susceptibility to phage infection, we selected for phage-resistant strains. Consequently, we isolated and sequenced the genome of an *L. biflexa* spontaneous mutant clone that was resistant to LE4 (Fig. 3A). This clone, called RLE4, was later screened by spot assays, which confirmed it as a true phage-resistant clone not only for LE4 but also for LE1 and LE3 (Fig. 3B). However, at a high concentration (10^5^ phage), LE4 is able to induce some cell lysis (Supplementary Fig. S4) probably due to the emergence of phages overcoming this resistance.

RLE4, as well as the parent susceptible strain, were sequenced on an Illumina platform. Mutations that were detected in sequenced strains were mapped against the *L. biflexa* reference genome (Fig. 3C). A single-nucleotide polymorphism (C->T at position 2,100,832) was identified. This mutation causes a premature stop codon in the gene *LEPBIa2019* which encodes a probable acetyltransferase. This nonsense mutation leads to a truncated protein of 30 kDa instead of 37 kDa for the native protein. Attempts to complement RLE4 with wild-type *LEPBIa2019* were unsuccessful (data not shown). Interestingly, *LEPBIa2019* is located in the *rfb*/O-antigen locus of the LPS that could be part of a large operon.

To confirm this initial observation, we generated a deletion mutant of *LEPBIa2019* by allelic exchange in *L. biflexa* (Fig. 3D) and tested for phage sensitivity. Spot assays demonstrated that the *LEPBIa2019* mutant was resistant to LE4 similarly to RLE4 (Supplementary Fig. S4), thereby validating that LE4 infectious cycle is O-antigen dependent as previously reported for other phages^22^. However, the adsorption of LE4 was not significantly reduced in the *LEPBIa2019* mutant (Supplementary Fig. S2), suggesting LEPBIa2019 gene product is not essential for viral adsorption under our experimental conditions, but may play a role in the entry of viral DNA into the bacterial cell. As shown in other phages, receptor requirements are complex and other cell surface components may play a role in adsorption and/or penetration of the phage^22^.

The host selectivity is probably determined by the specific interaction of the tail fibers of the phage with LPS of the host cell. An investigation of the O-antigen locus in 21 *Leptospira* species revealed that the susceptible strain *L. meyeri* 201601301 has an homologous region with *L. biflexa*, especially for the gene *LEPBIa2019* that shares an unusually high percentage (90.3%) of similarities between both species that is higher than percentages of similarities share with all the other species (Supplementary Fig. S5).

### Leptophage genome analysis

**Figure 4:**
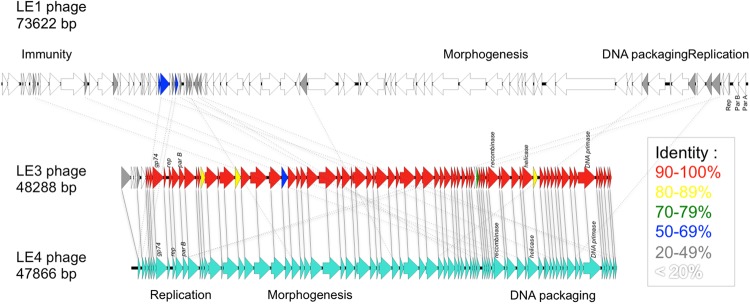
Linear genomic maps and protein identity comparisons between the leptophages. Based on nucleotide sequences of the phage genomes and predicted open reading frames. The direction of arrows represents the direction of transcription. Predicted functions and percent identity at the protein level are indicated.

To date, the 73,622-bp genome sequence of the temperate phage LE1 is the only genome of a phage infecting *Leptospira* to be available in public databases^8^. Here, we present the first complete genome sequences of lytic leptophages. The genomes of LE3 and LE4 are composed of 48,288 bp and 47,866 bp of double-stranded DNA, respectively, and have a GC content of 37.3%; similar to that of *L. biflexa* (38.6%) and LE1 (38.5%). In total, the genome of LE3 and LE4 had respectively 83 and 81 ORF, with all the ORF on the same strand (Fig. 4). Whole-genome sequence alignments reveal that the phages LE3 and LE4 are closely related (95% identity at the nucleotide level and 78 shared ORF), but are genetically distinct from LE1 (Fig. 4). In terms of gene repertoire, LE3 has only four genes encoding hypothetical proteins that do not have clear homologues in LE4 while three genes, also encoding for hypothetical proteins, are predicted to be unique to LE4. Given the high identity between LE3 and LE4, we decided to further focus our analysis on LE4. Of the 81 ORF of LE4, only 31 (38%) had a predicted function assigned according to BLASTp, PFAM or COG (Table 1). Accordingly, the majority of genes encode for proteins of unknown functions. Following the genome annotations, it was apparent that the phage genomes displayed a modular organisation which is common for the majority of tailed bacteriophage genomes such as LE1^8^. There are distinguishable gene modules whose products are predicted to be involved in DNA replication, structure/morphogenesis, and DNA packaging (Table 1, Fig. 4). It was not possible to identify genes encoding integrase, capsid proteins, or proteins involved in the process of host cell lysis (holins and lysins), further confirming that the majority of the phages’ genes remain uncharacterized.

**Table 1:** Annotation of predicted ORFs in LE4.

ORF	Length (aa)	Predicted function	Best BLAST hit	Organism	E-value	Identity	Conserved domains
1	88	replication	hypothetical protein	*Leptospira* phage LE1	9.0E-21	64%	
2	101	replication	hypothetical protein	*Leptospira* phage LE1	5.0E-11	43%	
3	312	replication	hypothetical protein	*Leptospira* phage LE1	9.0E-08	55%	
4	45						
5	56						
6	66						
7	95						
8	378		gp74	*Mycobacterium* phage Dori	9.0E-31	58%	PTZ00121 (MAEBL)
							pfam01991 (ATP synthase)
9	76						
10	102	replication	replication terminator protein	*Clostridium* phage phiCD111	1.0E-05	30%	
11	238						
12	179	replication	ParB-like nuclease domain containing protein	*Lactococcus* phage LW31	2.0E-07	30%	Cd16403 (ParB-like nuclease domain)
13	366	replication	hypothetical protein P12024S_24	*Persicivirga* phage	2.0E-42	35%	cd00267 (ATP-binding cassette transporter nucleotide-binding domain)
14	60						
15	75						
16	143		hypothetical protein SP010_00607	*Salmonella* phage FSL SP-010	4.0E-04	36%	
17	431	replication	terminase large subunit	*Lactococcus* phage Q33	9.0E-68	35%	cl17037 (Nucleotide-Binding Domain of the sugar kinase/HSP70/actin superfamily)
18	51						
19	505	structure-portal					pfam04860 (Phage portal protein)
20	194	structure					pfam11300 (Protein of unknown structure function)
21	308		ORF011	*Staphylococcus* phage 37	2.0E-03	32%	
22	548						
23	464						
24	214						
25	282	structure	head-to-tail connector protein	*Gordonia* phage Zirinka	9.2E-01	23%	
26	135						
27	199						
28	385						
29	592	structure-tail sheath	putative tail sheath protein	*Bacillus* phage BCD7	4.0E-10	26%	
30	141						
31	290						
32	71						
33	444	structure-tail tape	tape measure protein	*Gordonia* phage Lennon	9.0E-04	26%	COG5412 (Phage-related protein)
34	202		arginyl-tRNA synthetase	*Megavirus* courdo11	6.0E-06	23%	
35	85						
36	425						
37	242						
38	129						
39	506	structure-baseplate	putative baseplate J family protein	uncultured virus	4.0-08	25%	TIGR02243 (putative baseplate assembly protein)
40	221						
41	404	structure-tail	tail collar domain	*Sinorhizobium* phage phiLM21	1.0E-12	41%	COG5301 (Phage-related tail fibre protein)
							pfam07484 (Phage tail collar domain)
42	180	structure-tail fiber	tail fiber assembly-like protein	*Pseudomonas* phage phi3	2.0E-10	41%	pfam16778 (Phage tail assembly chaperone protein)
43	146						
44	222						
45	136						
46	93		hypothetical protein LEP1GSC193_0762	*Leptospira* phage vB_LalZ_80412-LE1	1.0E-04	30%	
47	122						
48	130						
49	87						
50	57						
51	63						
52	113						
53	78						
54	80						
55	69						
56	88						
57	93						
58	347	DNA recombination	recombinase	*Lactococcus* phage 49801	4.0E-18	35%	pfam03837 (RecT family)
							TIGR01913 (phage recombination protein Bet)
59	70						
60	302		hypothetical protein	uncultured Mediterranean phage uvMED	1.0E-39	32%	
61	271						
62	92						
63	356	DNA packaging	replicative DNA helicase	uncultured Mediterranean phage uvMED	3.0E-15	28%	cd00984 (DnaB helicase C terminal domain)
64	144						
65	41						
66	147		hypothetical protein SEA_TWISTER6_8	*Gordonia* phage Twister6	3.0E-14	29%	cd06554 (ASC-1 homology domain)
67	94		hypothetical protein	*Leptospira* phage LE1	3.0E-12	39%	
68	115		hypothetical protein CPT_Seuss95	*Caulobacter* phage Seuss	6.0E-16	43%	
69	140		ORF018	*Staphylococcus* phage 85	9.7E-01	45%	
70	76						
71	139						
72	263		hypothetical protein	uncultured Mediterranean phage uvMED	1.0E-47	50%	
73	149						
74	128						
75	577	replication	DNA primase	*Caulobacter* phage Sansa	2.0E-44	30%	pfam08275
							DNA primase catalytic core
							N-terminal domain
76	84						
77	75						
78	108						
79	101						
80	58						
81	60		hypothetical protein	*Leptospira* phage LE1	7.0E-15	51%	

### Proteomic analysis of LE3 and LE4

To further characterize LE3 and LE4 and identify major structure proteins, we decided to perform bottom-up proteomics experiments on purified phages prepared in biological replicates. A homemade protein sequence database composed of (i) the 83 and 81 different putative protein sequences deduced from LE3 and LE4 genomics data; (ii) the *L. biflexa* Uniprot database (UP000001847, 3723 entries); and (iii) usual proteomics contaminants was used for the search. We processed 2 to 3 replicates per phage and analyzed all 5 samples. The liquid chromatography/tandem mass spectrometry (LC-MS/MS) analysis reproducibly detected 8 and 11 proteins in LE3 and LE4, respectively (Supplementary Table 1). All the proteins found in LE3 were also present in LE4 confirming that the two phages are closely related. Among a total of 23 proteins found in LE4, the most abundant proteins, as expected, are structural proteins such as portal (gp19), head to tail connector (gp25), tail sheath (gp29 and gp33), tail collar (gp41), and baseplate (gp39) proteins. Other abundant proteins of unknown functions encoded by genes in the same locus, such as gp19 and gp22, may also belong to the same morphogenesis module. Surprisingly, no capsid protein was identified, suggesting that the capsid protein(s) in leptophages are highly divergent from other known capsid proteins. Three non-structural phage proteins, including proteins involved in DNA replication (gp12) and recombination (gp8, gp58), were also identified as part of the phage particle by mass spectrometry analysis, suggesting a possible contamination of the samples during the phage purification process. Alternatively, high-sensitivity LC-MS/MS may have lowered detection limits, making it possible to analyze not only structural proteins but also encapsidated enzymes.

Taken together, our data show that, even if bacteriophages are hugely abundant and crucial for various ecosystems^23^, their genomic sequences are underrepresented in public databases, comparatively to bacteria. This is particularly true for leptophages.

### Search of LE4-like prophages in *Leptospira* genomes

**Figure 5:**
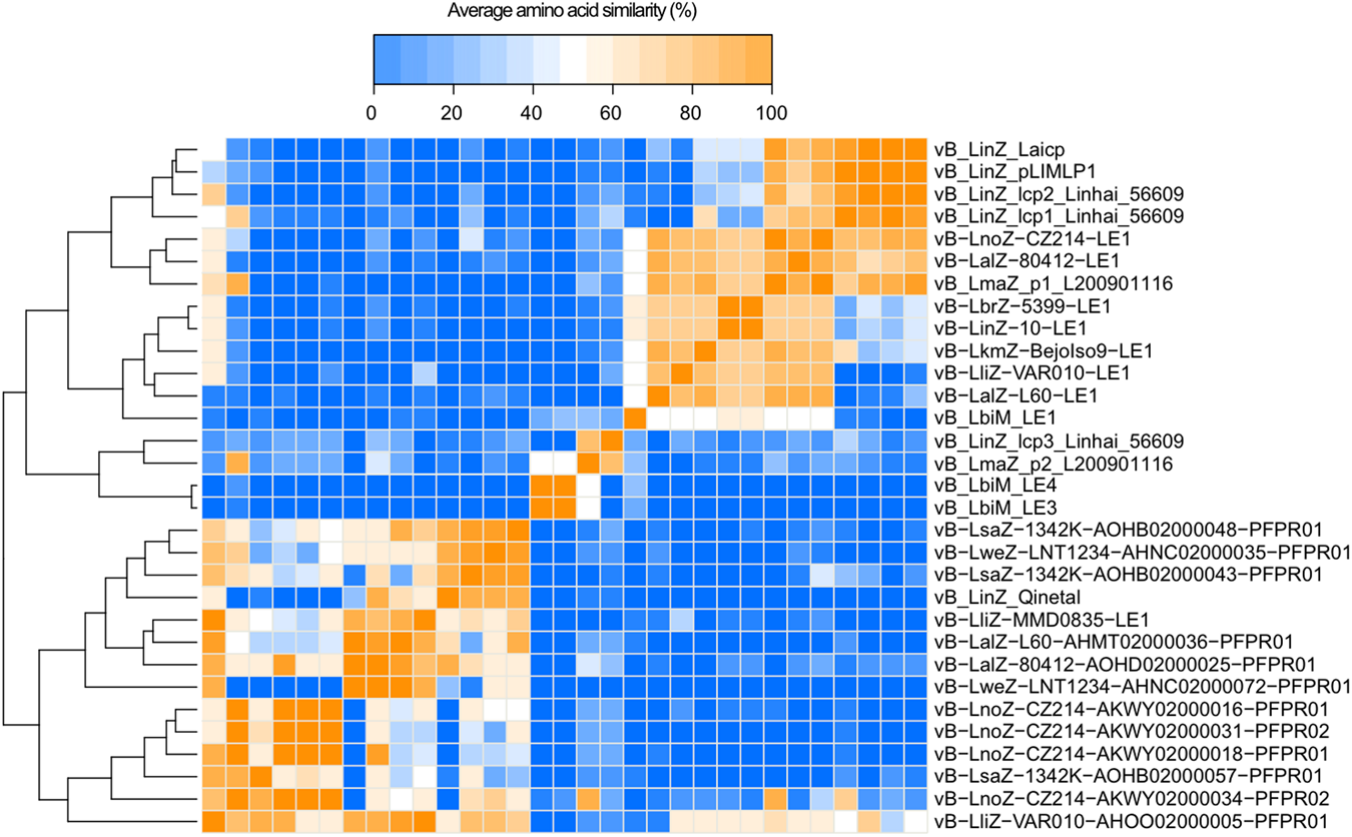
Clustering and heatmap of phage/prophage regions in *Leptospira* species. The colors represent, as indicated in the legend, the average similarity values for the 10 pairs of sequences having the highest bitcore values.

**Figure 6:**
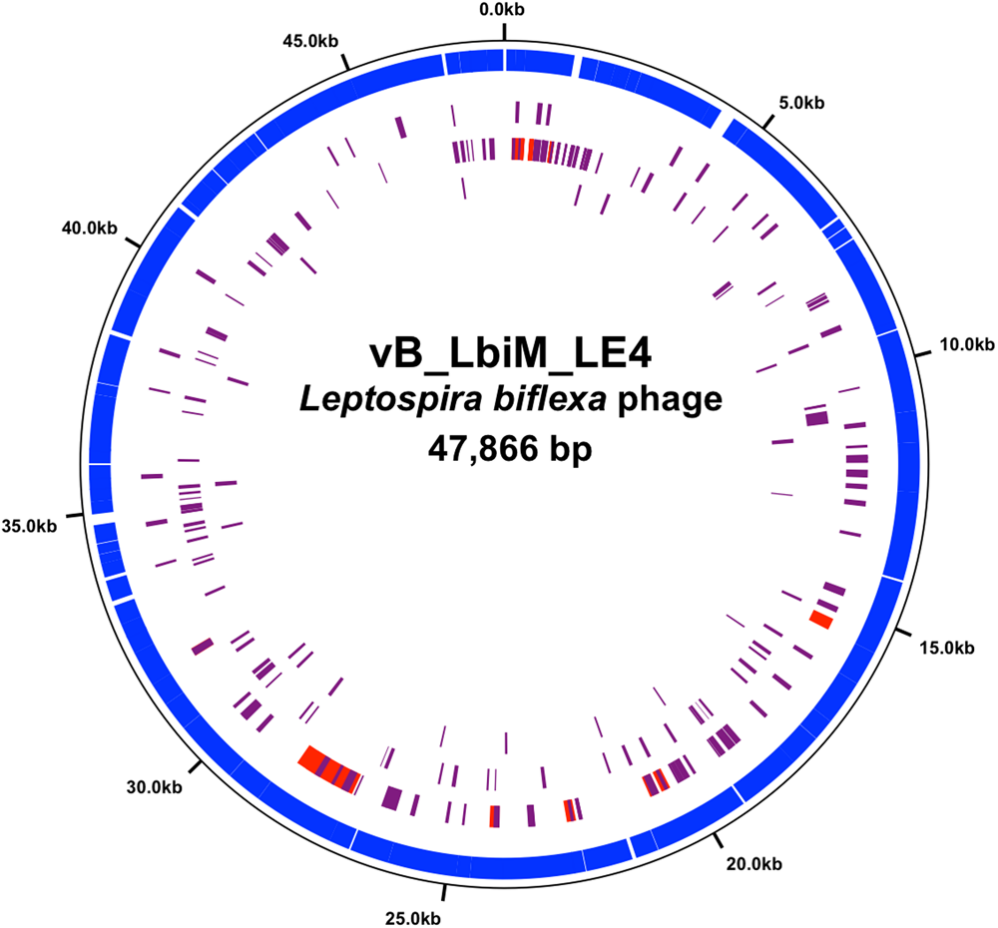
Circular genomic map and protein identity comparison between LE4, p2_L20090116 and lcp3. The circles represent from the outside to inside (1) LE4 CDS, and putative homologous loci with (2) p2_L200901116, (3) LE1 and (4) lcp3. The regions sharing bitscore values greater than or equal to 50 are in red while those sharing values inferior to 50 are in purple.

The phages LE3 and LE4 revealed strong DNA sequence identity, whereas no homologies to previously-known phage sequences were uncovered—even at the protein level. Comparison of the sequences of known or putative phages and prophages in *Leptospira* genomes suggested their partition into three main groups: the LE1-like (similar to the circular plasmid Laicp^14^), the Mu-like prophages^11^, and the LE4-like prophages. This latter group contains LE3, LE4, and two prophage-like regions in the pathogens *L. mayottensis* and *L. interrogans* (Figs 5 and 6). The LE4-like region in the pathogenic *L. mayottensis* strain 200901116 shares a strong synteny with the LE4 morphogenesis module and contains a phage-like replication origin (Fig. 4) that has been previously used to generate a replicative vector for pathogenic *Leptospira* strains^24^. A draft genome sequence of this strain with 84 contigs had been obtained previously from Illumina sequencing^25^. In an attempt to define all of the replicons present in *L. mayottensis* strain 200901116, the complete polished genome sequences were generated using the PacBio single-molecule real-time (SMRT) sequencing method, which is a powerful approach for discovering extrachromosomal replicons. The strain 200901116 consists of two circular chromosomes (3,815,263 bp in chromosome 1 and 307,019 bp in chromosome 2) and two circular plasmids of 94,000 bp (p1_L200901116) and 53,283 bp (p2_L200901116) exhibiting homologies to the LE1-like and LE4-like prophages, respectively. The LE4-like plasmid, p2_L200901116, has a GC content of 39.9% and contains 86 predicted CDS, with 74 CDS on the sense strand. Phage-related encoded proteins include a bacteriophage resistance factor (PF05565 family), phage terminase large subunit GpA-like protein, phage tail protein, phage tail tape measure protein, phage late control protein D, and phage tail collar protein. Again, genes encoding capsid proteins or lytic enzymes were not identified (Supplementary Table S2). At the protein level, 16 out of 86 predicted p2_L200901116 products are 49.76 ± 3.67% similar (29.66 ± 3.33% identical) to LE4 proteins. Plasmid p2_L200901116 also exhibits a high syntheny with the genes encoding putative structural proteins (gp19, gp21, gp22, gp23, gp28, gp29, gp31, gp33, gp34, gp36, gp37, gp40, and gp41) found in LE3 and LE4, suggesting that the morphogenesis module is conserved between the two phages.

A genetic relationship between LE3/LE4 and lcp3 was also established; lcp3 is a 55-kb circular plasmid from the pathogen *L. interrogans* serovar Linhai strain 56609 which produced tailed phage particles after induction with mitomycin C^10^. This plasmid, which contains genes encoding 30 phage-related proteins may, therefore, be a prophage-inducible plasmid of a lytic phage. In addition, another lcp3-related plasmid was also detected in *L. interrogans* serovar Australis strain 56607^10^. These phages (LE3 and LE4) and prophages (p2_L200901116 and lcp3) have a similarly sized (48–55 kb) circular genome and they likely share a common ancestor of lytic tailed phages.

In an attempt to induce the LE4-like prophage in *L. mayottensis* strain 200901116, cells were treated with mitomycin C, hydrogen peroxide and heat shock and then fixed for electron microscopy. We were not able to detect any phage particles in culture supernatants, suggesting that the prophage induction may be sensitive to other stimuli such as signals encountered *in vivo* during host-pathogen interactions. Alternatively, p2_L200901116 is defective for phage growth as a consequence of DNA rearrangements or other factors.

## Concluding Remarks

In summary, the present study suggests that *Leptospira* possess an unknown diversity of phages, of which most have no homologues with in public databases. LE3 and LE4 are genetically distinct from the temperate phage LE1. *Leptospira* genome sequencing revealed LE4-like prophages existing as circular plasmids in the pathogens *L. interrogans* and *L. mayottensis*. We suggest that LE4 is the founder of a new group of phages, to which LE3, lcp3, and p2_L200901116 also belong. This study is also the first to demonstrate that LPS is a receptor for leptophages and suggests that LPS structure dictates host succeptibility.

Prophages play an important role in the genetic evolution and diversification of bacteria, including the emergence of virulence^26,27^. Since prophages are ubiquitous in the genomes of pathogenic *Leptospira* strains^11^, it is likely that they have contributed to the adaptation of pathogens to their hosts. Further sequencing of phage genomes will advance several aspects of the field including detection methods for prophages in *Leptospira* genomes, development of genetic tools (such as phage-based DNA delivery systems), identification of novel genes, prediction of definitive or putative functions of hypothetical proteins, and better understanding of the phages contribution to bacterial evolution and virulence.

## Methods

### Bacterial strains and growth media

*Leptospira biflexa* serovar Patoc strain Patoc 1 was isolated from Parisian stream water^28^ and maintained in the collection of the National Reference Centre for Leptospirosis (Institut Pasteur, Paris, France). *L. mayottensis* strain 200901116 was isolated from a patient with leptospirosis in Mayotte, Indian Ocean^25^. *L. meyeri* strain 201601301 was isolated from stream water in Mayotte. *Leptospira* strains were grown at 30 °C in liquid Ellinghausen, McCullough, Johnson and Harris (EMJH) medium^29^.

### Bacteriophages

Phages LE1, LE3 and LE4 were isolated from sewage water in Paris, France^7^. Titration of phages was done in EMJH 1.2% agar medium with an EMJH 0.6% agar overlay. For phage isolation, 400 mL of an exponential phase culture of *L. biflexa* strain Patoc 1 (DO_420nm_ = 0.1) was infected with 10^6^ PFU of phages (MOI = 0.001), then incubated at room temperature for 30 days. The phage lysate was centrifuged for 30 min at 7,000 g and the supernatant was supplemented with 8% PEG 8000 and 0.3 M NaCl and incubated overnight at 4 °C as previously described^30^. The phage solution was centrifuged for 30 min at 7,000 g. The supernatant was discarded and the pellet was re-suspended in 10 mL Difco™ *Leptospira* Medium Base EMJH. To remove contaminants, 20% chloroform was added, then eliminated by centrifugation for 30 min at 1700g, and the phage lysate was filtered through a 0.1 μm syringe filter. Phages were purified by isopycnic gradient centrifugation in 45% caesium chloride (38,000 rpm, 24 h, 4 °C; Beckman Coulter rotor SW41) as previously described^31^.

### Phage absorption and one-step growth kinetics

For phage adsorption assays, *L. biflexa* strain Patoc 1 and *LEPBIa2019* mutant cultures were infected with LE4 at MOI of 0.0001 at room temperature and at different times after infection (0 min, 30 min, 1 h, 2 h, 3 h and 6 h), the number of unadsorbed phages remaining in the supernatant following immediate filtration through a 0.22 µm filter was determined by plating. Briefly, 100 µL of the filtrate was incubated with 100 µL of a culture of 10^8^ bacteria per mL for 30 min then phage titration was determined by the double-layer agar plate method^7^, expressed as plaque-forming units per milliliter (PFU/mL).

One step growth experiments were done as previously described^32^. Following an adsorption period of 30 min at a MOI of 10, samples were obtained at 2 hours intervals, and immediately filtrated and plated for phage titration determined by the double-layer agar plate method as described above. The burst size was determined relative to the initial number of phages (t = 0).

### Isolation of phage-resistant mutants and lysogeny verification

A liquid culture of *L. biflexa* infected with LE4 (MOI = 0.001) was incubated for 30 days, and then the lysed culture, still showing several viable *Leptospira* as revealed by dark-field microscopy, was inoculated into fresh EMJH liquid medium. Colonies of the phage-resistant strain were isolated from an EMJH plate and the colonies were confirmed to resist phage infection by spot assays (described below). A total of 22 resistant clones were isolated in three independent experiments and all were tested by PCR assay using primers targeting LE4 sequence (LE4_F2 5′-ATGCGGAATGTCTTGAAGGC-3′ and LE4_R 5′-TGTCAAGGATGCGGTAGGTT-3′) to detect potential lysogeny. We then selected one phage-resistant mutant strain, called RLE4, for subsequent analysis.

For targeted mutagenesis, a spectinomycin resistance cassette replacing the coding sequence of *LEPBIa2019* and flanked by 0.9 kb sequences homologous to the sequences flanking the target gene was synthesized by GeneArt (Life Technologies, Grand Island, NY, USA) and cloned in a pMK-RQ *E. coli* vector, which is not able to replicate in *Leptospira*. Allelic exchange was obtained by transforming the UV-pretreated suicide plasmid by electroporation as previously described^9^ with a Biorad Gene Pulser Xcell™.

Spot assays of both *L. biflexa* WT, RLE4, and *LEPBIa2019* mutant strains were conducted as three independent replicates to determine the strain susceptibility to phage infection. One millilitre of an exponential phase culture (DO_420nm_ = 0.1) was spread onto EMJH plates and, one hour later, drops containing 10^2^, 10^3^, or 10^4^ PFU of LE1, LE3, or LE4 were spotted on lawns and then incubated at 30 °C for 4 days.

### Electron Microscopy

Bacteriophages were allowed to adsorb onto a carbon-coated copper grid and negatively stained with 4% uranyl acetate^33^. Grids were observed under an FEI Tecnai T12 Transmission Electron Microscope (TEM) with an acceleration voltage of 120 kV and at a magnification of 49,000. To observe phage infection and host cell lysis, we used a MOI of 10 and a 2% glutaraldehyde fixation was performed prior negative staining; non-infected cells were used as controls.

### Mass spectrometry

Mass spectrometry analysis was performed on LE3 and LE4 (titres of 10^9^ PFU/mL and 10^10^ PFU/mL, respectively) purified by caesium chloride density gradient centrifugation. In order to get rid of BSA contained in the EMJH medium, a step of depletion was performed using a BSA Depletion Column according to the manufacturer protocol (IgY Kit, GenWay Biotech, San Diego, CA, USA). To remove contamination by PEG 8000 used during purification a 10 kDa filtration step was performed using an Amicon Ultra-0.5 ml Ultracel-10k (Millipore). After reduction and alkylation, proteins were digested by 0.5 μg of Sequencing-Grade Modified Trypsin (Promega, Madison, WI, USA) overnight at 37 °C according to the eFASP protocol^34^.

Tryptic peptides were analyzed on a Q Exactive Plus instrument (Thermo Fisher Scientific, Bremen) coupled with an EASY nLC 1200 chromatography system (Thermo Fisher Scientific). Five µL was loaded on an in-house packed 50 cm nano-HPLC column (75 μm inner diameter) with C18 resin (1.9 μm particles, 100 Å pore size, Reprosil-Pur Basic C18-HD resin, Dr. Maisch GmbH, Ammerbuch-Entringen, Germany) and equilibrated in 97% solvent A and 3% solvent B (ACN, 0.1% FA). Peptides were first eluted using a 3 to 22% gradient of solvent B for 112 min, then a 22 to 38% gradient of solvent B for 35 min and finally a 38 to 56% gradient of solvent B for 15 min all at 250 nL/min flow rate. The instrument method for the Q Exactive Plus was set up in the data dependent acquisition mode. After a survey scan in the Orbitrap (resolution 70,000), the 10 most intense precursor ions were selected for HCD fragmentation with a normalized collision energy set up to 27. Charge state screening was enabled, and precursors with unknown charge state or a charge state of 1 and >7 were excluded. Dynamic exclusion was enabled for 45 s. All data were searched using Andromeda with MaxQuant software^35^ version 1.5.3.8. Five amino acids were required as minimum peptide length and 1 unique peptide was required for protein identification. A false discovery rate (FDR) cutoff of 1% was applied at the peptide and protein levels.

### Genome sequencing

Phages purified by a caesium chloride gradient were dialysed in *Leptospira* Medium Base EMJH (dialysis cassette 2000 MWCO, Thermo Fisher). Phage solutions were treated with DNase and RNase, and EDTA was used to terminate the reactions. Phage DNA was then extracted using the QIamp Minelute virus spin kit (Qiagen) and amplified with Repli-g mini kit (Qiagen). For whole genome sequencing of *L. biflexa* Patoc strains, DNA was extracted using a MagNA Pure 96 Instrument. Next-generation sequencing was performed by the Mutualized Platform for Microbiology (P2M) at Institut Pasteur, using the Nextera XT DNA Library Preparation kit (Illumina), the NextSeq 500 sequencing systems (Illumina), and the CLC Genomics Workbench 9 software (Qiagen) for analysis.

Genomic DNA of *L. mayottensis* strain 200901116 was extracted with the Genomic tip 100 g kit (Qiagen) from a 35 ml culture according to manufacturer protocols and the complete genome sequence was obtained using SMRT (Pacific Biosciences) technology. PacBio sequencing was performed at the Génome Québec Innovation Centre (McGill University, Montreal, Canada) using a Pacific BioScience RS II system. The sequencing reads were de novo assembled using HGAP^36^ through SMRT Analysis.

### Bioinformatics analysis

Genome annotation was performed with the MaGe interface (https://www.genoscope.cns.fr/agc/mage)^37^. LE3 and LE4 sequences are available in this interface. Similarity searches were performed using BLASTp^38^ (with a BLASTp e-value of 1 × 10^−3^ as a cut-off) and conserved domains. ORF denomination in Table 1 and Supplementary Table S1 correspond to MaGe nomenclature.

We compared the Single-nucleotide polymorphisms (SNPs) of mutant RLE4 against the wild-type parental strain. Sequence reads were aligned with the annotated *L. biflexa* serovar Patoc strain Paris I genome (accession numbers CP000786, CP000787 and CP000788^39^) by using the Burrows-Wheeler Alignment tool (BWA mem 0.7.5a)^40^. SNP calling was done with the Genome Analysis Toolkit Unified Genotyper (GATK 2.7-2)^41^ by following Broad Institute best practices. Candidate SNPs were further filtered by requiring coverage of greater than half of the genome mean coverage and 95% read agreement to validate the call. SNPs, short indels, and coverage were visualized with SynTView^42^. The data are accessible through the following link: http://hub18.hosting.pasteur.fr/SynTView/flash/Leptospira/Leptospira_biflexa.html. This finding was confirmed by sequencing the PCR product of this region amplified with primers Pat2019-F (5′-GATGGAACATGATCCAACTA-3′) and Pat2019-R (5′-GAATTGAATGGTCTTGGCACA-3′).

Average similarity values were computed using the 10 hits having the highest bitscore values (blastp argument of DIAMOND^43^) for all combinations of phages presented in Fig. 5. Since some (pro)phages do not share 10 ORF, the average similarities values were multiplied by (n/10), where n is the number of shared ORF. The resulting square matrix was clustered using heatmap.2 from the statistical package R^44^. The map of LE4 and the tblastx results presented in Fig. 6 were visualized using circleator version 1.0.0^45^.

### Nucleotide sequence accession number

The LE1 bacteriophage genome accession number is BX571876. The nucleotide sequences of LE3 and LE4 bacteriophages have been deposited in the GenBank database as accession numbers MF974396 and MF974397, respectively. The sequence of *Leptospira mayottensis* strain 200901116 was deposited in GenBank under the accession number CP024871 (chromosome I), CP024872 (chromosome II), CP024873 (plasmid p1_200901116), and MF974398 (plasmid p2_200901116).

## Electronic supplementary material

Supplementary figures S1, S2, S3, S4, S5

Supplementary tables S1, S2
