# Supplementary material for: Characterization of LE3 and LE4, the only lytic phages known to infect the spirochete Leptospira
Source: Sci Rep. 2018 Aug 6;8:11781. doi: 10.1038/s41598-018-29983-6 (PMC6078989; doi:10.1038/s41598-018-29983-6)
Supplement: Supplementary file 1 — Supplementary figures S1, S2, S3, S4, S5 [file 41598_2018_29983_MOESM1_ESM.pdf]

Supplementary figures for

Characterization of LE3 and LE4, the only lytic phages known to infect the spirochete *Leptospira*

Olivier Schiettekatte[1], Antony T. Vincent[2], Christian Malosse[3], Pierre Lechat[4], Julia Chamot-Rooke[3], Frédéric J. Veyrier[2], Mathieu Picardeau[1] & Pascale Bourhy[1]

[1] Institut Pasteur, Unité Biologie des Spirochètes, Paris, France. [2] INRS-Institut Armand-Frappier, Bacterial Symbionts Evolution, Laval, Quebec, Canada. [3] Institut Pasteur, Citech, Mass Spectrometry for Biology Utechs, USR 2000 IP CNRS, Paris, France. [4] Institut Pasteur, Bioinformatics and Biostatistics Hub, C3BI, USR 3756 IP CNRS, Paris, France.

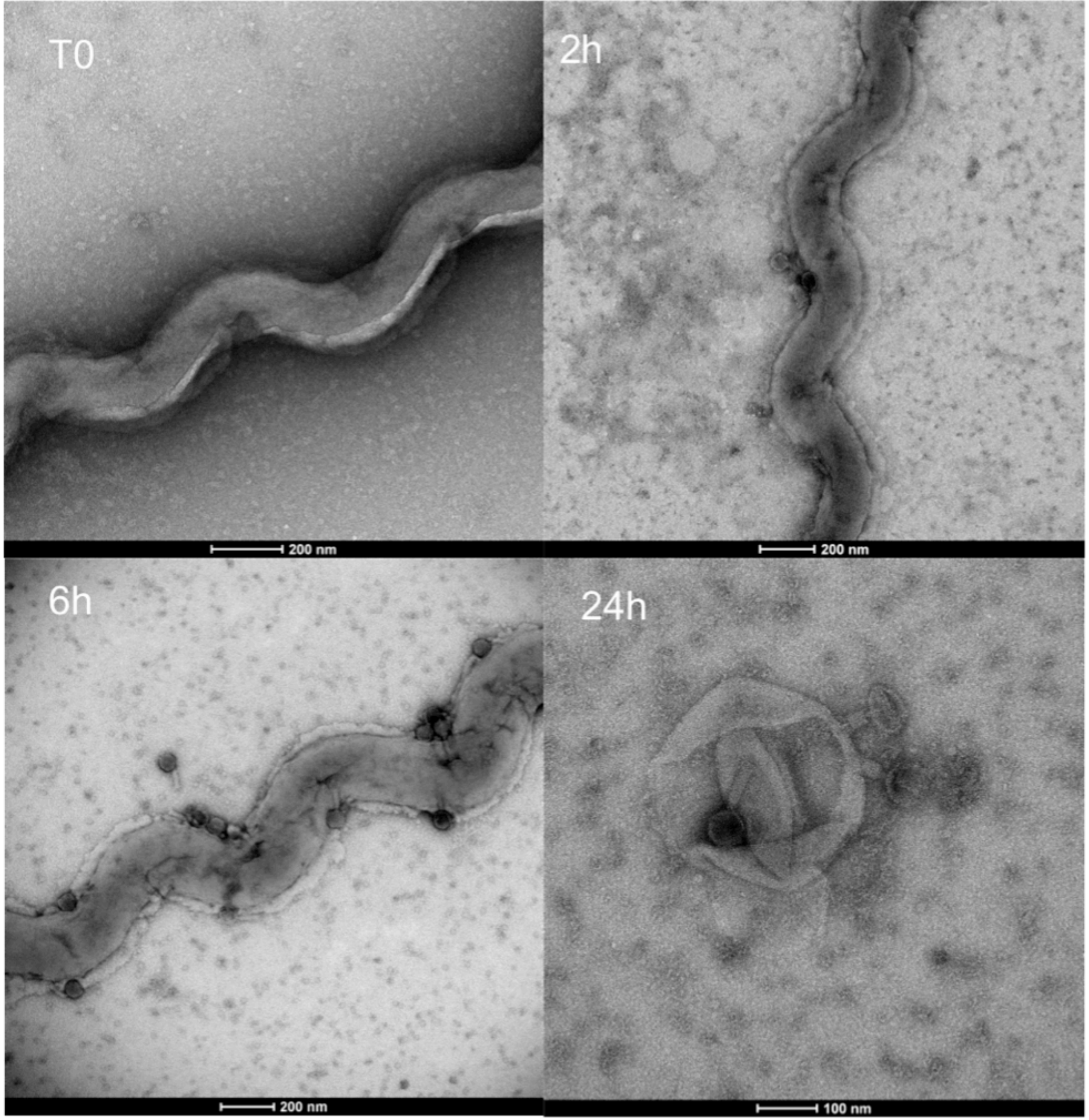

Supplementary Figure S1. Micrographs of *L. biflexa* (total cell length of ~ 10µm) infected with phage LE4 (MOI=10) at t = 0, 2h (adsorption), 6h (release), and 24h (lysis) after infection.

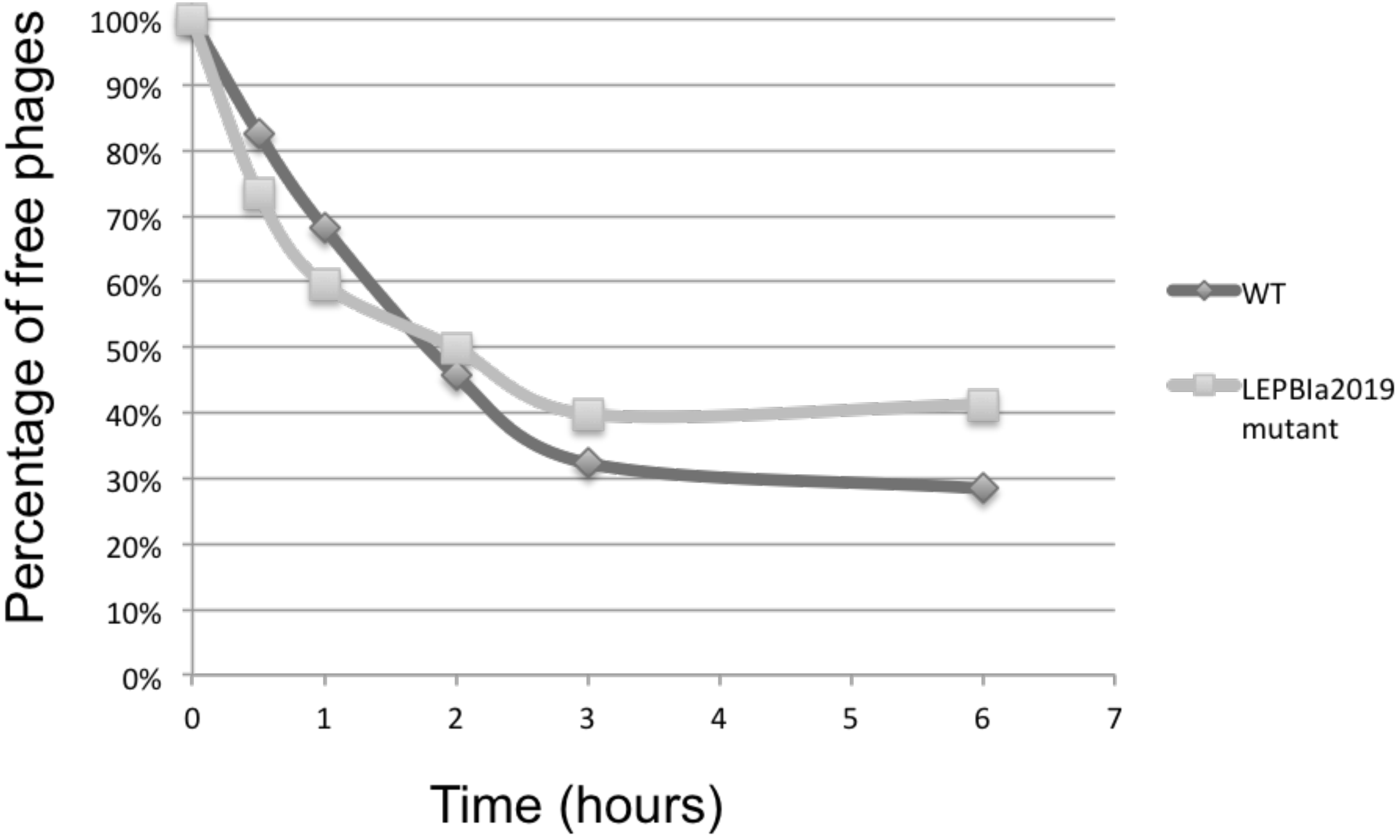

Supplementary Figure S2. Adsorption kinetics of phage LE4 on WT and *LEPBIa2019* mutant *L. biflexa* (mean of 3 independent experiments)

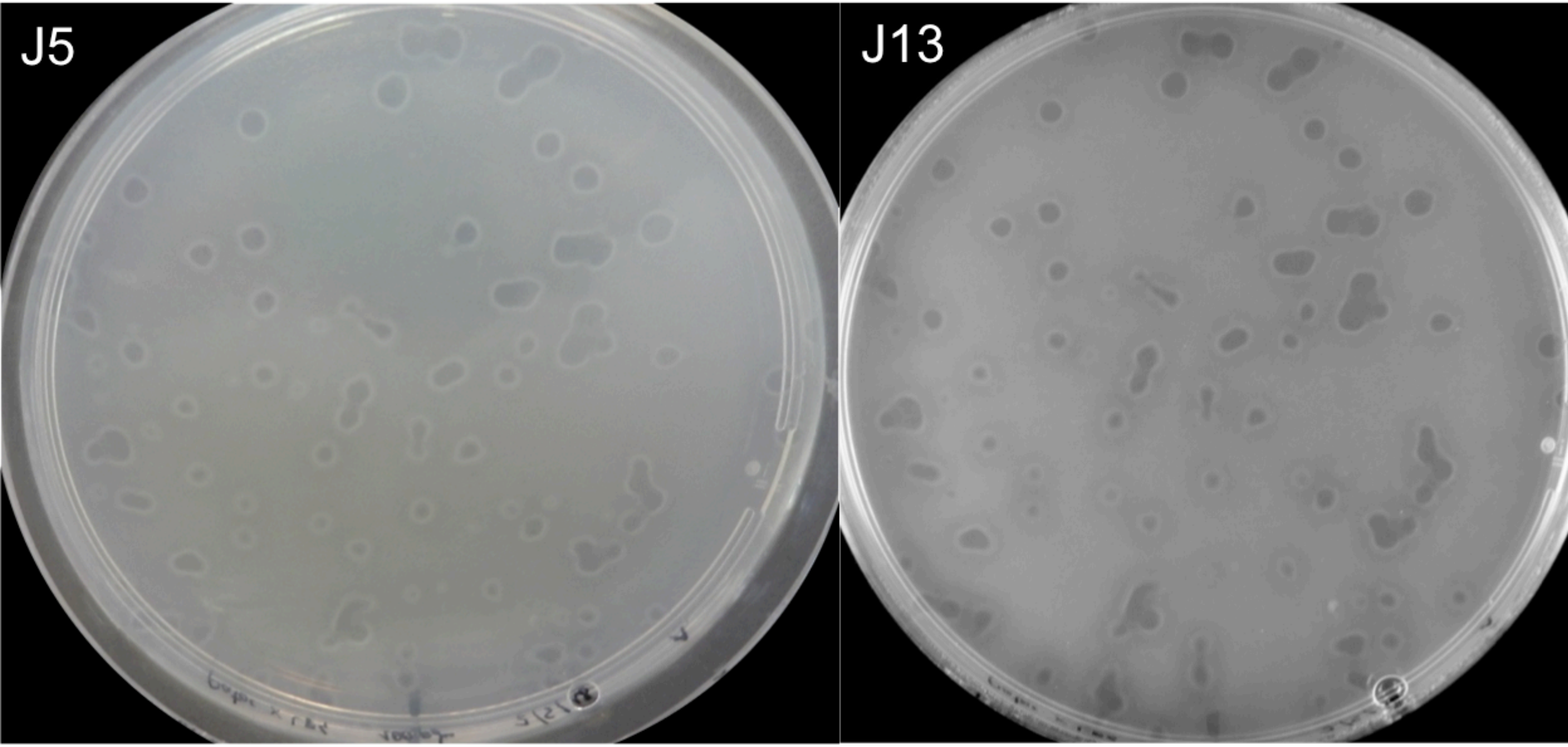

Supplementary Figure S3. Comparison of LE4 lysis plaques pattern (99 pfu) after 5 and 13 days of incubation.

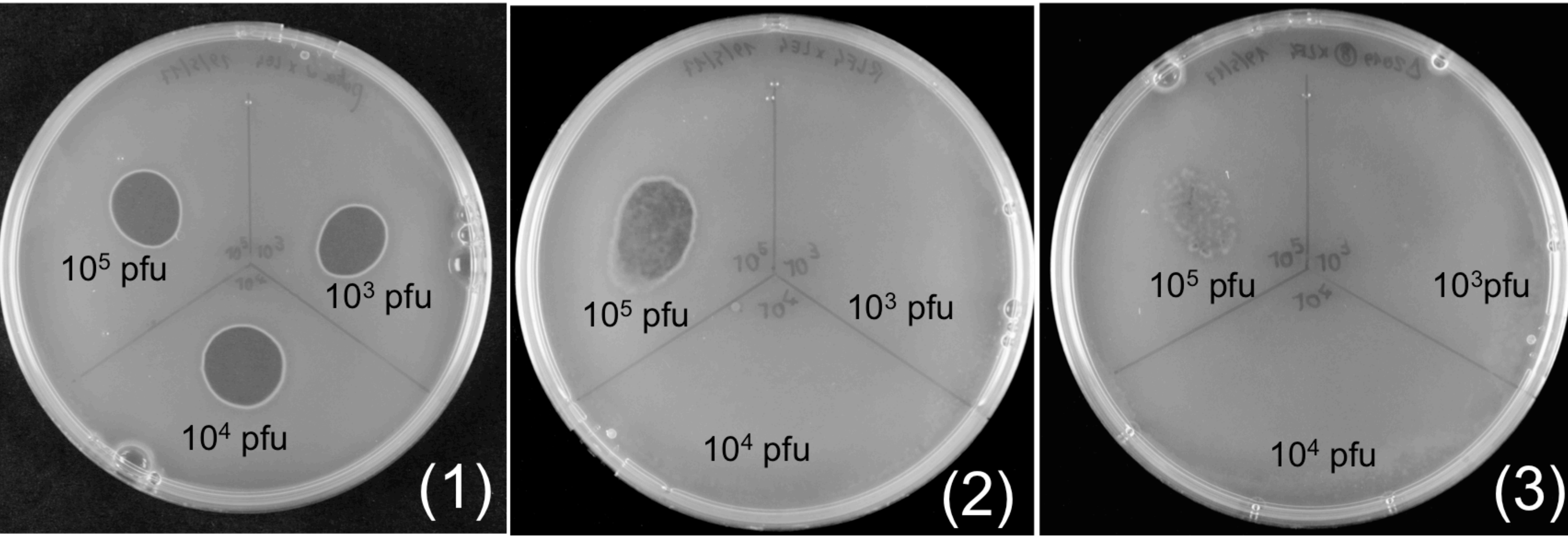

Supplementary Figure S4. Spots assays of LE4 against the WT strain (1), the RLE4 resistant strain (2), and the LEPBIa2019 mutant (3)

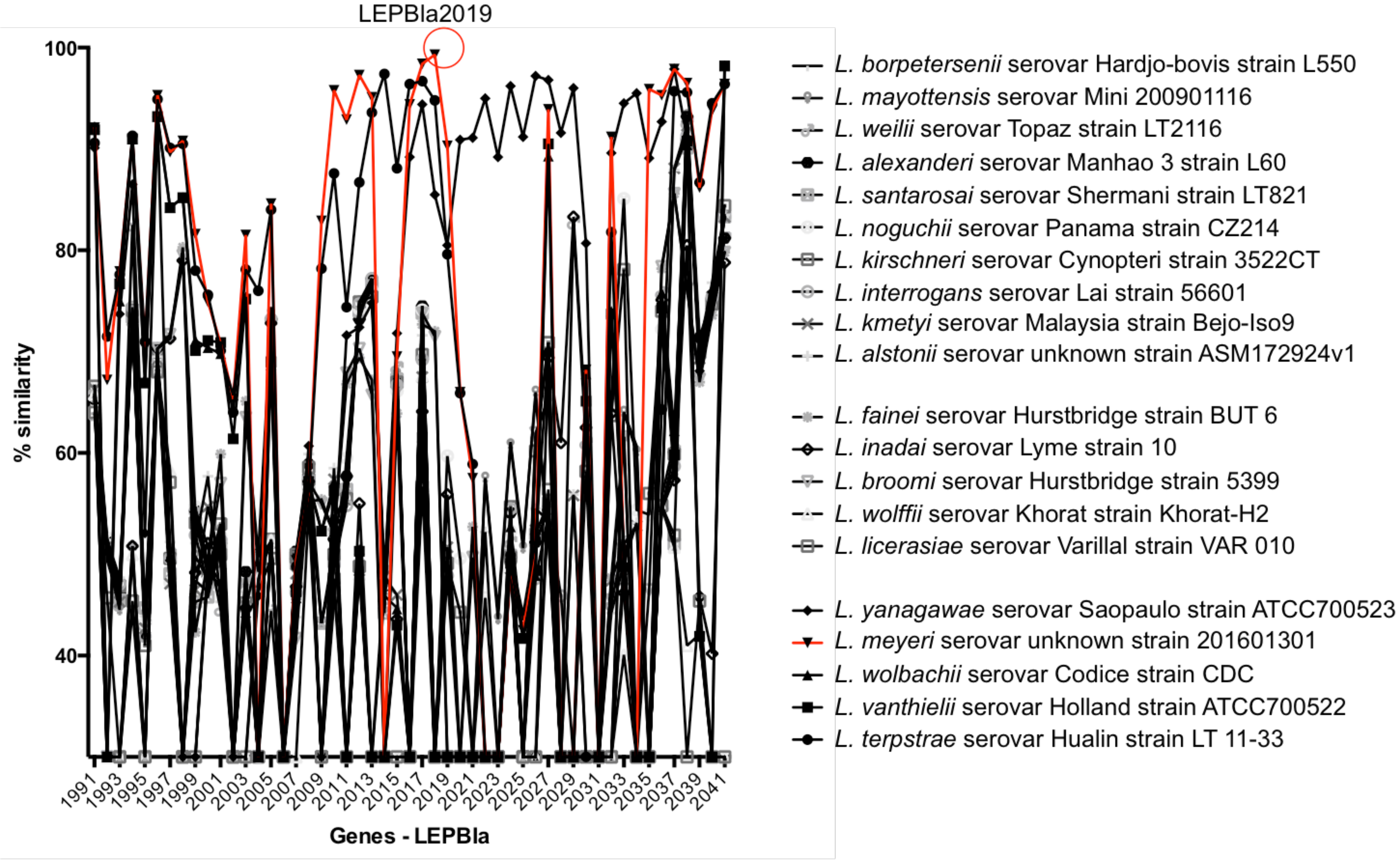

Supplementary Figure S5. Percentage of similarity of the O-antigen locus from *L. biflexa* LPS compared with the 21 sequences *Leptospira spp.*
